# PK/PD modeling supports the dose-escalation decision in VIKING

**DOI:** 10.1186/1758-2652-13-S4-P182

**Published:** 2010-11-08

**Authors:** M Lovern, M Underwood, G Nichols, I Song

**Affiliations:** 1 GlaxoSmithKline, 5 Moore Drive, Research Triangle Park, USA

## 

In the VIKING study a 50 mg once daily regimen of the next generation HIV integrase inhibitor (INI) S/GSK1349572 (572) exhibited promising antiviral activity in an initial cohort (Cohort I) of treatment-experienced subjects with documented raltegravir (RAL) resistance. The purpose of this work was to evaluate via simulation techniques whether improved virologic responses could be achieved with higher doses in a second cohort (Cohort II) by utilizing pharmacokinetic (PK) and pharmacodynamic (PD) models derived using Cohort I data.

Interim PK data from VIKING Cohort I were combined with data from Phase 1 and 2a studies in healthy and HIV+, INI-naïve subjects, respectively; data were analyzed using a linear two-compartment PK model. The population PK/PD analysis incorporated log10 HIV-1 RNA viral load (VL) sampled throughout 10 days of dosing during the Phase 2a and VIKING studies. The VL was modelled using an indirect response model in which the 572 plasma concentrations inhibited HIV-1 RNA production. Final PK and PK/PD models were validated using the visual predictive check (VPC) technique. Two sets of simulations were used to predict responder percentage for dose regimens proposed for Cohort II of VIKING. First, change from baseline in log_10_ VL (ΔVL) at Day 11 was simulated for cohorts of 1000 subjects for each dose regimen according to different fixed levels of baseline fold change (FC) in 572 IC50 from wild type virus. The second set of simulations predicted responder percentages for clinical RAL-resistant HIV populations with diverse 572 susceptibilities. The data were well-described by the respective models. Model parameters were generally well-determined and VPC plots verified predictive performance.

Simulations predicted increasing the dose regimen from 50 mg once daily to 50 mg twice daily would increase the percentage of patients with FC=8 that achieved ≥ 1.5 log_10_ ΔVL at Day 11 by ~28%. Similarly, improvements in response of ~20% and ~18% were predicted for patient populations with HIV resistance profiles observed in RAL PhIIb and BENCHMRK virologic failure and VIKING screening populations, respectively. Our models predict 572 50mg twice daily will appreciably increase Day11 virologic responses in RAL-resistant subjects, supporting the dosing strategy for the ongoing Cohort II. 572 shows promise to demonstrate further the activity in this difficult to treat patient population.

